# Influence of gingival phenotype on crestal bone loss at implants: A long-term 2 to 20-year cohort study in periodontally compromised patient

**DOI:** 10.1186/s40729-024-00531-4

**Published:** 2024-08-13

**Authors:** Nicole Breunig, Michael Stiller, Martin Mogk, Reiner Mengel

**Affiliations:** 1 Department of Prosthetic Dentistry, School of Dental Medicine, Philipps-University Marburg/Lahn, Georg-Voigt Str. 3, 35039 Marburg, Lahn, Germany; 2 moreDATA GmbH, Gießen Kerkrader Strasse 11, 35394 Gießen, Germany

**Keywords:** Dental implants, Periodontal disease, Ginigival phenotyp, Crestal bone loss

## Abstract

**Purpose:**

The aim of this long-term cohort study in periodontally compromised patients with implants was to analyze the correlation between gingival phenotype and peri-implant crestal bone loss, and between clinical measures and gingival phenotype.

**Methods:**

Implant-supported single crowns and bridges were used to rehabilitate 162 implants in 57 patients. Patients were examined over a 2 to 20-year period on a recall schedule of 3 to 6 months. In addition to recording clinical parameters, intraoral radiographs were taken at baseline (immediately after superstructure insertion) and at 1, 3, 5, 10, 15, and 20 years. Patients were differentiated into phenotype 1 with thin, scalloped gingiva and narrow attached gingiva (*n* = 19), phenotype 2 with thick, flat gingiva and wide attached gingiva (*n* = 23), or phenotyp 3 with thick, scalloped gingiva and narrow attached gingiva (*n* = 15).

**Results:**

The mean peri-implant crestal bone loss during the first 12 months was 1.3 ± 0.7 mm. Patients with gingival phenotype 1 had a significantly greater rate of increased crestal bone loss at implants (*p* = 0.016). No significant differences were present in subsequent years. The prevalence of mucositis at all implants was 27.2%, and the prevalence of peri-implantitis 9.3%. Univariate analyses indicated a significantly higher peri-implantitis risk in patients with gingival phenotype 2 (p-OR = 0.001; p-OR = 0.020). The implants of patients with phenotype 2 had significantly greater probing depths (1st year *p* < 0.001; 3rd year *p* = 0.016; 10th year *p* = 0.027; 15th year *p* < 0.001). Patients with gingival phenotype 3 showed no significantly increased probing depths, signs of inflammation and crestal bone loss.

**Conclusions:**

Patients with a gingival phenotype 1 have greater crestal bone loss at implants during the first year of functional loading. Patients with gingival phenotype 2 had significantly greater probing depth at implants and risk of peri-implantitis.

## Introduction

Crestal bone loss at implants during the first 12 months after loading is based on an initial physiological bone remodeling process after surgical trauma and functional loading of the crestal bone [1]. In subsequent years, annual bone loss (≤ 0.2 mm) may occur, even at healthy implants [2]. Peri-implant bone loss progresses via microbially associated, host-mediated inflammation and non-inflammatory factors, such as osseous pathologies, poorly controlled diabetes, titanium particle release, and occlusal overloading [3].

The quality and quantity of peri-implant soft tissue may also influence crestal bone loss. Clinical studies have shown that the width of the keratinized mucosa is important in peri-implant health, with positive associations between crestal bone loss and lack of keratinized tissue, especially when inflammation is present [4–6]. A narrow band of keratinized mucosa is associated with plaque accumulation, inflammation, and mucosal recession, which may promote crestal bone loss.

Currently, only short-term studies have correlated local soft tissue thickness at the implant site with peri-implant crestal bone loss. Systematic reviews have shown that implants surrounded by thin soft tissue suffer from higher rates of crestal bone loss during the first 12 months of functional loading [7, 8]. However, the importance of the local soft tissue in maintaining the health of the peri-implant tissue remains unclear [9]. Furthermore, it is difficult to compare studies because the peri-implant soft tissue was evaluated in different studies according to different criteria.

World Workshop 2017 aimed to establish uniform definitions and methods to enable comparability and improve the validity of future clinical studies [10, 11]. Clinically, periodontal soft tissue at teeth should be differentiated according to the gingival and periodontal phenotype. Gingival phenotype describes the three-dimensional soft tissue volume, which comprises the soft tissue thickness, scalloping gingiva, and width of the attached gingiva. Periodontal phenotype is defined as the combined gingival phenotype and bone morphotype (i.e., buccal bone plate thickness). Currently, no long-term clinical study has investigated the influence of the gingival phenotype on crestal bone loss at implants.

The objective of this long-term cohort study in periodontally compromised patients with implants was to analyze the correlation between gingival phenotype and peri-implant crestal bone loss. We also analyzed the correlation between gingival phenotype and clinical parameters at implants.

## Materials and methods

### Study population

Fifty-seven patients (40 females and 17 males, age 20 to 65 years, mean age at implant insertion: 48.2 years) with periodontitis were treated at the School of Dental Medicine, Philipps University, Marburg/Lahn. A 3-month recall schedule followed the systematic periodontal treatment for 4 to 6 years. A detailed description of the periodontal treatment procedure was published previously [12, 13].

Before implant placement, all patients had healthy periodontal tissue with no bleeding on probing (BOP) and probing depth (PD) < 3 mm. The eligibility criteria for participating in the present study were evaluated between 1992 and 2017. The inclusion/exclusion criteria were described in detail previously [12, 13].

Patients were classified as stages I-IV according to the AAP/RFP definition based on periodontal disease severity and progression [14], specifically the interproximal radiographic bone loss. Stage was determined by the worst parameter or site. If the bone loss at the most severe site (mesial or distal) was < 15%, it was designated stage I, 15–33% was designated stage II, and > 33% was stage III/IV. The diagnosis was adjusted for complexity using a pocket depth ≥ 6 mm, the presence of an intrabony defect ≥ 3 mm deep, and the presence of furcation (degree II or III). Grade A, B, or C was added to the diagnosis based on the ratio of the percentage bone loss radiographically to patient age. If the ratio was < 0.25, it was designated grade A, 0.25–1.0 was designated grade B, and > 1.0 was designated grade C. We determined the disease extent as the number of teeth with clinical attachment loss divided by the total number of teeth; generalized disease was defined as > 30% and localized disease as < 30%. Regarding periodontal disease severity, 20 patients were classified as stage I and 37 patients as stage II. Referring to the progression of periodontal disease, 29 patients were classified as grade A and 28 patients as grade B.

Patient treatment was carried out in accordance with the Declaration of Helsinki by the World Medical Association (version VI, 2002). This study was approved by the Ethics Committee Marburg/Lahn (ek_mr_11_07_2017_ mengel).

### Implant placement and prostheses

A total of 162 dental implants were inserted epicrestally: 45 with a smooth surface (Brånemark® Mk II and III, Nobel Biocare, Zurich, Swiss) and 117 with a rough surface (Nobel Replace® Straight Groovy and Nobel Speedy® Replace, Nobel Biocare, Zurich, Swiss). The implants were 8.5 to 15.0 mm in length and had a diameter of 3.75 to 5.0 mm (Table 1). All implant placements were prosthetically driven using pre-fabricated surgical stents. The bone quality and quantity were classified during insertion of the implant [15]. Twenty-one patients received 1 implant, 18 patients received 2 or 3 implants, and 18 patients received 4 or more implants. In 8 patients, the deficient buccal bone walls of 15 implants were augmented during insertion with autologous bone obtained from the surgical site and xenogenic bone substitute material (Geistlich; BioOss Spongiosa, Wolhusen, Swiss). The implants and augmented materials were covered by a xenogenic membrane (Geistlich; BioGide, Wolhusen, Swiss).

**Table 1:** Patients and implants

		total	phenotype 1	phenotype 2	phenotype 3
**Patients**					
**Gender**	male	17	5	8	4
	female	40	14	15	11
**Implants**		162	55	61	46
**Surface**	smooth	45	12	20	13
	rough	117	43	41	33
**Topography**	maxilla	80	30	35	15
	mandibular	82	25	26	31
**Augmentation**	yes	15	2	10	3
	no	147	53	51	43
**Bone quality**	type 1	4	2	0	2
	type 2	146	52	51	43
	type 3	12	1	10	1
**Bone quantity**	A	8	2	2	4
	B	103	36	44	23
	C	36	8	10	18
	D	15	9	5	1
**Superstructure**	single crown	123	47	40	36
	bridges (*n* = 19)	39	8	21	10
**Retention**	screw-retained	37	12	10	15
	cemented	125	43	51	31

Second-stage surgery was performed after 3 or 6 months in the mandible and maxilla, respectively. No additional connective tissue or epithelial grafts were applied. Implant insertion and second-stage surgeries were performed according to the manufacturer’s guidelines by the same periodontist (RM). Four weeks after second-stage surgery, the patients were treated with fixed single crowns (*n* = 123) or bridges (*n* = 19). Screw-retained (*n* = 37) or cemented (*n* = 125) restorations were used based on the clinical situation and preference of the clinician. All bridges and crowns consisted of a high-gold metal framework veneered with ceramic or full ceramics. Prosthetic treatment was performed at the Dental School of Medicine, Philipps University, Marburg/Lahn.

### Follow-up examination

The first clinical examination was carried out immediately after the final superstructure was inserted and considered to be baseline. Subsequently, patients were treated for 2 to 20 years on a 3 to 6-month recall schedule (Table 2). A detailed description of the treatments in the recall was published previously [12, 13]. All patients were in the recall program for at least 2 years. In addition, 53 of the patients (150 implants) were followed for 5 years, 35 patients with 96 implants for 10 years, 21 patients with 39 implants for 15 years, and 9 patients with 18 implants for 20 years.

**Table 2:** Number of patients and implants during observation period

Years	Patients	Implants
≥ 2	57	162
≥ 3	56	157
≥ 4	55	155
≥ 5	53	150
≥ 6	50	143
≥ 7	46	135
≥ 8	43	120
≥ 9	38	110
≥ 10	35	96
≥ 11	32	88
≥ 12	31	84
≥ 13	28	65
≥ 14	22	43
≥ 15	21	39
≥ 16	19	37
≥ 17	17	33
≥ 18	13	24
≥ 19	11	20
20	9	18

### Clinical examination

A periodontal probe (UNC-15; Hu-Friedy, Chicago, IL, USA) was used for clinical measurements at six sites (buccal, mesiobuccal, distobuccal, lingual, distolingual, mesiolingual) for each tooth and implant. We also investigated the plaque index (PI) [16], gingival index (GI) [17], PD (in mm), and BOP (in %). Clinical examinations were performed by experienced examiners (*n* = 10) calibrated for intra-examiner (correlation coefficients 0.98 to 0.98) and inter-examiner reproducibility (correlation coefficients 0.96 to 0.97). Routine calibration sessions in which a minimum of 50 sites were measured in duplicate in at least five patients were scheduled every 12 months.

### Gingival phenotype

The gingival phenotype of each patient was determined at the central anterior teeth of the maxilla. If these teeth were prosthetically restored or missing, the lateral anterior teeth were used, otherwise we used the canines. All patients had to have at least four natural teeth in the maxillary anterior region at the time the gingival phenotype was determined.

The soft tissue thickness was measured centrally on the buccal side of the tooth using a standard periodontal probe (DB765R, Hu-Friedy, Chicago, IL, USA). The probe was single-ended and color-coded with black markings (1 to 15 mm) and #30 handling. To accomplish this, we observed the periodontal probe by transparency through the gingival tissue after insertion into the gingival sulcus at a depth of 1 mm [18]. We used the visibility of the tip to classify the gingival thickness: thin if the tip was visible, thick if the tip was not visible. All assessments were performed without any magnification in natural light. The oral cavity was not illuminated to avoid light scattering or interference in observing the gingival transparency.

The contour of the gingival margin was determined by the papilla height mesially and distally to the central maxillary anterior teeth. For this purpose, the periodontal probe (DB765R, Hu-Friedy, Chicago, IL, USA) was placed perpendicular to the junction line between the buccal sulcus up to the papilla tip to assess papilla formation [19]. A complete papilla, which filled the proximal space completely, presented with a scalloped gingival margin, whereas incomplete papilla presented with a flat gingival margin.

**Fig. 1:**
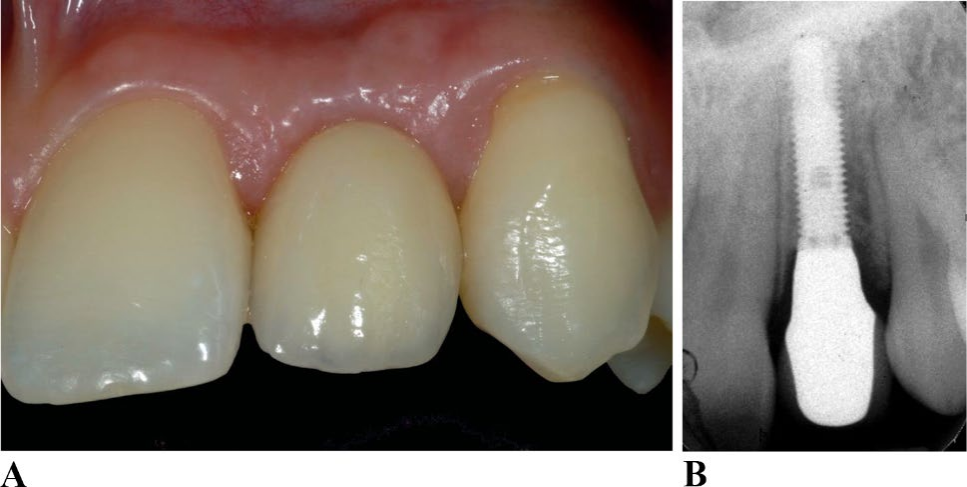
**a** Implant lateral incisor 5 years of insertion of crown. Gingival phenotype 1 with thin, scalloped gingiva and narrow attached gingiva. **b** Intra-oral radiograph 5 years of insertion of crown

The width of the attached gingiva was measured mid-buccally at teeth using the periodontal probe (DB765R, Hu-Friedy, Chicago, IL, USA). The evaluation represents the distance (in mm) between the mucogingival junction and free gingival margin. The alveolar mucosa was stretched several times to identify the mucogingival junction. A narrow attached gingiva had a width of ≤ 2 mm and a wide attached gingiva had a width > 2 mm. The gingival phenotype was categorized into three classes based on the thickness of the gingiva, scalloped gingiva, and width of the attached gingiva:

(a) Gingival phenotype 1 (thin-scalloped gingival phenotype) with thin, scalloped gingiva.

and narrow attached gingiva. (Figure 1a and b)

**Fig. 2:**
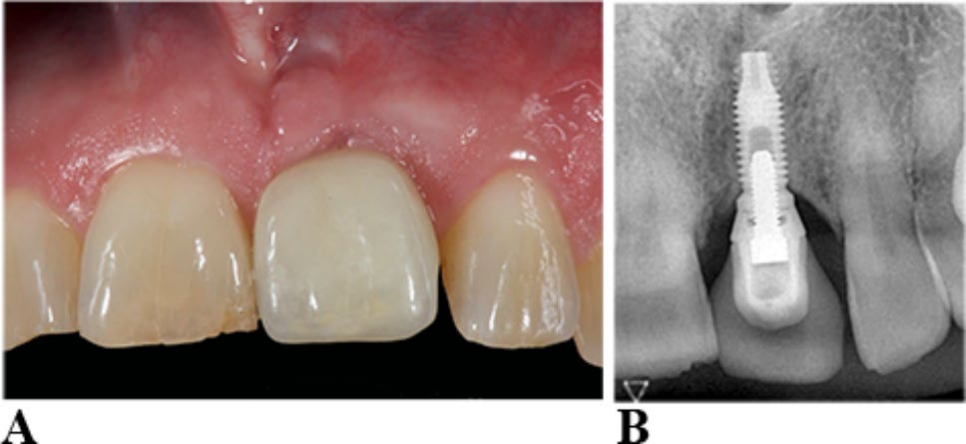
**a** Implant frontal incisor 12 years of insertion of crown. Gingival phenotype 2 with thick, flat gingiva and wide attached gingiva. **b** intra-oral radiograph 12 years of insertion of crown

(b) Gingival phenotype 2 (thick-flat gingival phenotype) with thick, flat gingiva and wide.

attached gingiva. (Figure 2a and b)

**Fig. 3:**
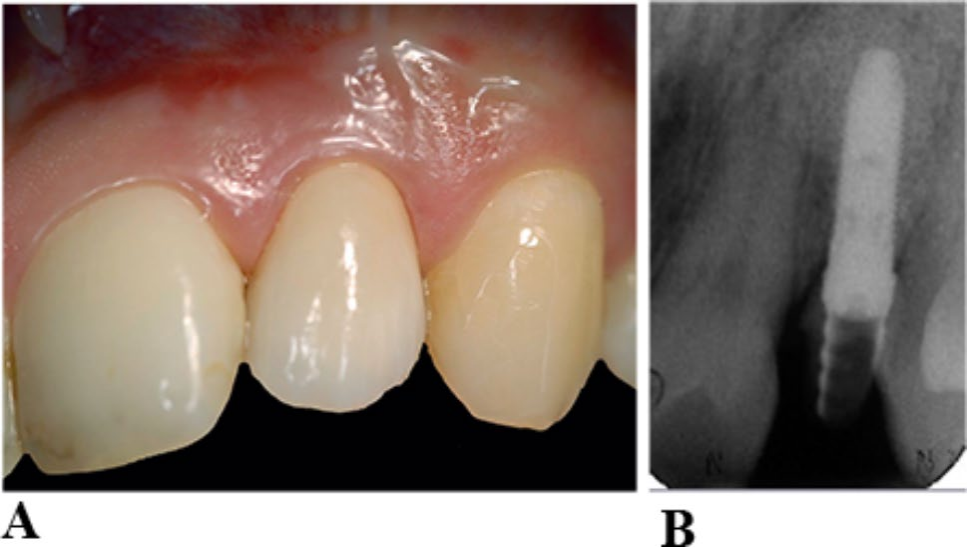
**a** Implant lateral incisor 8 years of insertion of crown. Gingival phenotype 3 with thick, scalloped gingiva and narrow attached gingiva. **b** intra-oral radiograph 8 years of insertion of crown

(c) Gingival phenotype 3 (thick-scalloped gingival phenotype) with thick, scalloped gingiva and narrow attached gingiva. (Figure 3a and b)

The clinical measurements for gingival phenotype were performed by an experienced and trained examiner (NB) calibrated for intra-examiner reproducibility as described above for clinical examinations.

### Radiographic measurement

Standardized intra-oral radiographs were taken of all implants by experienced radiologists using a parallel long-cone technique with Rinn-holders (XCP Instruments, Rinn Corporation Elgin, IL, USA). Customized individual bite registration was not used. Radiographs were obtained at baseline (immediately after superstructure insertion) and then after 1, 3, 5, 15, and 20 years. Initially, each analog radiograph was framed as a slide and digitized at a resolution of 675 dpi using a SnapScan slide scanner (Agfa, Mortsel, Belgium). However, images were taken digitally (Planmeca, Helsinki, Finland) after 2005. All digitized radiographs were evaluated by an independent masked examiner using computer software (Planmeca Romexis, Helsinki, Finland). Before measurement, the intra-examiner reproducibility was calibrated as noted above for other examinations. The radiograph was calibrated by the implant length or the thread of the implant. If the threads were not clearly visible on the radiograph and calibration was not possible, the radiograph was not used. At implants, the distance from the first apical contact between bone and implant to the implant abutment connection was measured mesially and distally (in mm) and related to the implant thread.

### Statistical analysis

All data for all patients were stored in the NBImplant database of the Coordination Center for Clinical Studies (KKS), Philipps University, Marburg/Lahn. Statistical analyses of the clinical and radiographic results were performed using the program R for Windows version 3.4.1. The implants were the evaluation units of the statistical analyses. Normal Q-Q plots and the Shapiro-Wilk test indicated a non-normal data distribution, so nonparametric methods were used for statistical analyses. The implant survival rate was determined by Kaplan-Meier analysis. The survival rates of the three subgroups were compared by the log-rank test. The alpha level of the study was *p* = 0.05. Clinical parameters were analyzed for all implants together and separately according to gingival phenotype. To compare the means between the gingival phenotypes, a nonparametric Kruskal-Wallis test was used. The Dunn test was used as a multiple comparisons test. Significant levels were corrected by Bonferronie Holm. The Spearmen non-parametric correlation analysis was used to analyze the correlation of clinical parameters with increased crestal bone loss (BLI > 0.1 mm/year) compared with the measured value from the previous year. Qualitative classification of the correlation into effect sizes was achieved using the Cohen analysis.

The diagnosis of peri-implant diseases during the entire study period was determined by clinical and radiological results [12]. We used a peri-implant mucositis definition of PD ≥ 5 mm with BOP or GI ≥ 2 without annual bone loss, whereas peri-implantitis was defined as PD > 5 mm with or without BOP or GI ≥ 2 and annual bone loss > 0.2 mm. This definition of peri-implantitis requires an annual determination of bone loss and results in a more accurate determination of bone loss than with the current AAP/RFP definition [20].

## Results

None of the patients were lost to follow-up, and we followed all patients for at least 2 years (Table 2). None of the patients were smokers or regularly consumed alcohol, and the patients had no systemic diseases. All of the patients regularly attended the scheduled follow-up appointments and had excellent oral hygiene.

During the observation period, one implant had to be removed after 5 years due to implant fracture. Thus, the implant survival rate was 99.3% after 20 years. The following complications occurred throughout the study period: chipping of the ceramic veneer (*n* = 12), abscess/suppuration (*n* = 3), and loosening of the superstructure (*n* = 7). Because of the low significance due to the small number of technical and biological complications, a statistical analysis was not meaningful.

The classification of patients according to gingival phenotype revealed 19 patients with phenotype 1 (33.3%), 23 patients with phenotype 2 (40.4%), and 15 patients with phenotype 3 (26.3%; Table 2). The distribution of implants by gingival phenotype revealed 55 implants in patients with gingival phenotype 1 (33.9%), 61 implants in patients with gingival phenotype 2 (37.7%), and 46 implants in patients with gingival phenotype 3 (28.4%).

In the first year after insertion of the final superstructure (baseline), the mean PD of implants was 2.56 ± 0.88 mm, which increased to 3.25 ± 0.61 mm after 20 years. The comparison of PD between the three gingival phenotypes showed significantly increased PD at implants in patients with phenotype 2 (1st year *p* < 0.001; 3rd year *p* = 0.016; 10th year *p* = 0.027; 15th year *p* < 0.001).

The mean BOP of all implants was 0.13 ± 0.21 in the first year and increased continuously over 20 years to 0.28 ± 0.23 (Table 3). The comparison of BOP showed significantly increased BOP at implants in patients with gingival phenotype 2 in the third year (*p* = 0.001). The mean GI of implants was 0.32 ± 0.55 in the first year, decreasing over 20 years to 0.19 ± 0.38 (Table 4). The comparison of GI showed a significantly increased GI for implants in patients with gingival phenotype 2 (*p* = 0.004).

**Table 3:** Bleeding on Probing (BOP) at implants

Year	Total	Phenotype 1	Phenotype 2	Phenotype 3	Kruskal- Wallis- test	Dunn-test
1	0,13 ± 0,21	0,05 ± 0,11	0,18 ± 0,26	0,14 ± 0,20	*p* = 0,055	-
3	0,14 ± 0,18	0,07 ± 0,10	0,22*±0,24	0,10 ± 0,11	*p* = 0,001	1 vs. 2 *p* < 0,001
5	0,16 ± 0,18	0,16 ± 0,19	0,16 ± 0,18	0,15 ± 0,16	*p* = 0,916	-
10	0,17 ± 0,17	0,18 ± 0,16	0,20 ± 0,19	0,13 ± 0,12	*p* = 0,319	-
15	0,20 ± 0,15	0,23 ± 0,14	0,21 ± 0,15	0,17 ± 0,17	*p* = 0,376	-
20	0,28 ± 0,23	0,29±0,31	0,30 ± 0,21	0,20 ± 0,15	*p* = 0,669	-

Mean BOP with standard deviation in mm

p = significance value (Kruskal- Wallis- test and Dunn- test)

*= significantly increased mean

**Table 4:** Gingival Index (GI) at implants

Year	Total	Phenotype 1	Phenotype 2	Phenotype 3	Kruskal- Wallis- test	Dunn- test
1	0,32 ± 0,55	0,15 ± 0,36	0,47 ± 0,71	0,20 ± 0,35	*p* = 0,323	-
3	0,45 ± 0,50	0,47 ± 0,51	0,48 ± 0,51	0,36 ± 0,48	*p* = 0,731	-
5	0,37 ± 0,48	0,38 ± 0,44	0,33 ± 0,50	0,41 ± 0,50	*p* = 0,587	-
10	0,17 ± 0,26	0,16 ± 0,20	0,19 ± 0,30	0,11 ± 0,20	*p* = 0,570	-
15	0,17 ± 0,32	0,00 ± 0,01	0,23*±0,34	0,18 ± 0,39	*p* = 0,004	1 vs. 2 *p* < 0,013
20	0,19±0,38	0,00±0,00	0,28±0,46	0,04±0,08	*p*=0,259	-

Mean GI with standard deviation in mm

p = significance value (Kruskal- Wallis- test and Dunn- test)

*= significantly increased mean

In all patients, the mean crestal bone loss at implants approximatley 12 months after functional loading was 1.30 ± 0.70 mm. In subsequent years, slight crestal bone loss was present at almost all implants, with up to 1.70 ± 0.80 mm after 20 years. We found no significant differnces in the mean crestal bone loss at implants between patients with the three gingival phenotypes.

To compare crestal bone loss between implants, whether crestal bone loss increased (BLI > 0.1 mm/year) compared to the previous year was determined for each implant (Table 5). Overall, 64 ± 39% of implants from patients with gingival phenotype 1 had increased crestal bone loss (*p* = 0.016) in the first year of functional loading. No further significant differences were observed during subsequent years.

**Table 5:** Percentage of implants with increased crestal bone loss (BLI > 0.1 mm/year)

Year	Phenotype 1	Phenotype 2	Phenotype 3	Kruskal- Wallis- test	Dunn- test
1	64%*±39%	36%±38%	25 ± 38%	*p* = 0,016	1 vs. 3 *p* = 0,025
3	50%±46%	57%±45%	58%±40%	*p* = 0,862	-
5	37%±44%	36%±41%	35%±39%	*p* = 0,990	-
10	24%±35%	14%±24%	20 ± 29%	*p* = 0,558	-
15	14%±21%	13%±19%	13%±22%	*p* = 0,986	-
20	14%±22%	31%±26%	12%±21%	*p* = 0,187	-

Percentage of implants with increased crestal bone loss (BLI > 0.1 mm) with standard deviation

p-= significance value (Kruskal- Wallis- test and Dunn- test)

*= significantly increased percentage of implants with increased crestal bone loss

Over the entire observation period, the correlation of clinical parameters with increased peri-implant crestal bone loss (BLI > 0.1 mm annually) was investigated (Table 6). A low significant correlation for PI (*p* = 0.040, *R* = 0.156) and a low negative correlation for the width of the keratinized mucosa (*p* = 0.001, *R*=-0.253) was found for all implants regardless of gingival phenotype. Correlation analysis by gingival phenotype showed a moderately significant correlation for mucosal recession (*p* = 0.047, *R* = 0.329) and a small negative correlation for PD (*p* = 0.024, *R*=-0.235), with increased crestal bone loss at implants in patients with gingival phenotype 1. For the implants of patients with gingival phenotype 2, a small negative correlation of the width of the keratinized mucosa (*p* = 0.016, *R*=-0.261) with increased crestal bone loss was observed.

**Table 6:** Correlation of clinical parameters, gingival phenotype and increased crestal bone loss (BLI > 0.1 mm/year)

	PD	BOP	PI	GI	MR	AM
Total	*R*= -0.076	*R*= -0.088	R = 0.156*	*R*= -0.078	*R* = -0.024	R = -0.253*
	(*n* = 341)	(*n* = 293)	(*n* = 174)	(*n* = 216)	(*n* = 235)	(*n* = 164)
	(*p* = 0.164)	(*p* = 0.132)	(*p* = 0.040)	(*p* = 0.254)	(*p* = 0.717)	(*p* = 0.001)
Phenotype1	R= -0.235*	*R*= -0.172	*R*= -0.105	*R*= -0.171	R = 0.329**	*R* = -0.323
	(*n* = 92)	(*n* = 72)	(*n* = 32)	(*n* = 33)	(*n* = 37)	(*n* = 32)
	(*p* = 0.024)	(*p* = 0.149)	(*p* = 0.566)	(*p* = 0.341)	(*p* = 0.047)	(*p* = 0.072)
Phenotype2	*R* = 0.011	*R*= -0.082	*R* = 0.177	*R*= -0.076	*R* = -0.084	R = -0.261*
	(*n* = 141)	(*n* = 134)	(*n* = 90)	(*n* = 124)	(*n* = 130)	(*n* = 85)
	(*p* = 0.902)	(*p* = 0.344)	(*p* = 0.096)	(*p* = 0.404)	(*p* = 0.340)	(*p* = 0.016)
Phenotype3	*R*= -0.083	*R*= -0.079	*R* = 0.257	*R*= -0.061	*R* = -0.103	*R* = -0.235
	(*n* = 108)	(*n* = 87)	(*n* = 52)	(*n* = 59)	(*n* = 68)	(*n* = 47)
	(*p* = 0.394)	(*p* = 0.465)	(*p* = 0.066)	(*p* = 0.648)	(*p* = 0.405)	(*p* = 0.111)

*= to illustrate the low significant correlation effect according to Cohen

**= to illustrate the mean significant correlation effect according to Cohen

R = correlation coefficient

n = number of measurements

p = significance value

PD = Probing depth

BOP = Bleeding on probing

PI = Plaque index

GI = Gingival index

MR = mucosal recession

AM = Width of keratinized mucosa

Further significant differences and correlations between the gingival phenotype, gender, periodontal staging and grading, implant surface, implant topography, bone structure, bone quality and quantity, prosthetic restoration, and fixation type were not found.

At all implants, the prevalence for peri-implant mucositis was 27.2% and peri-implantitis 9.3% (Table 7). Univariate analyses of the occurrence of peri-implant diseases showed a significantly higher risk for patients with gingival phenotype 2 in terms of peri-implantitis (p-OR = 0.001; p-OR = 0.020).

**Table 7:** Prevalence of peri-implantitis

	Peri-implantitis (*n*=15 cases)					95% CI			
Parameter		yes	no	total	percentage	OR	min	max	p-OR
Gender	female	105	11	116	9.5%	1			
	male	37	4	41	9.8%	1.032	0.226	3762	1
Phenotype	1	53	1	54	1.9%	1			
Phenotype 2 vs. 1	2	45	13	58	22.4%	15.031	2.105	660.537	0.001*
Phenotype	1	53	1	54	1.9%	1			
Phenotype 3 vs. 1	3	44	1	45	2.2%	1.202	0.015	96.241	1
Phenotype 2 vs. 1	2	45	13	58	22.4%	1			
Phenotype 3 vs. 2	3	44	1	45	2.2%	0.078	0.009	2.400	0.020*
Topography	maxilla	66	11	77	14.3%	1			
	mandible	76	4	80	5%	0.318	0.07	1.138	0.059

CI = confidence intervall; OR = Odds-Ratio; min = minimum; max = maximum;

*= significant p-OR

## Discussion

The importance of the width of the keratinized mucosa in regard to peri-implant health has been investigated in many clinical studies, which have shown that an adequate soft tissue cuff is provided at implant sites by the band of keratinized mucosa [21, 22]. Few clinical studies have correlated local soft tissue thickness at the implant site with peri-implant crestal bone loss [23–25]. A prospective study in periodontally healthy patients with single-tooth bone-level implants (Straumann, Basel, Swiss) measured the thickness of the peri-implant soft tissue after crestal incision with a periodontal probe to distinguish between thin (≤ 2 mm) or thick (> 2 mm) soft tissue [23]. At 1 year, significantly less crestal bone loss had occurred around implants placed in thick mucosal tissue. These results could not be confirmed in a prospective 1-year study comparing clinical measures and crestal bone loss around tissue-level implants between patients with thin and thick vertical mucosa at the edentulous site in the mid-crestal region [26]. The results showed that the soft tissue thickness does not affect crestal bone loss at implants after the first 12 months of loading. In a systematic review based on six clinical studies, four studies showed significantly higher initial crestal bone loss at implants placed at sites with soft tissue thickness < 2 mm, and no significant difference was found in two studies [7]. However, none of the included studies had of low risk of bias. These contradictory results on the influence of the thickness of the local soft tissue on crestal bone loss at implants show that many questions are still unanswered.

No clinical study has yet to determine the effect of gingival phenotype on crestal bone loss at implants. Due to the long observation period, the present study provides new insights into the correlation between gingival phenotype, clinical parameters, and crestal bone loss. The results in periodontally compromised patients with thin, scalloped gingiva and narrow attached gingiva (gingival phenotype 1) indicate significantly greater crestal bone loss at implants during the first 12 months of functional loading. Furthermore, patients with thick, flat gingiva and wide attached gingiva (gingival phenotype 2) had significantly greater PD at implants and risk of peri-implantitis. No increased risk of soft tissue recession and peri-implantitis was observed in individuals with thin gingiva and narrow attached gingiva. These results at implants only partially confirm the correlation between clinical parameters at teeth and gingival phenotype [27]. Patients with thick gingiva are more prone to an increased PD at teeth. In contrast, patients with thin gingiva and narrow attached gingiva have an increased risk of periodontal changes, such as recession, inflammation, and periodontitis. Because of the heterogeneity of the results, additional clinical studies are required to demonstrate the possible differences in long-term stability between the soft tissue around teeth and implants.

The effect of periodontal disease history on the correlation of gingival phenotype with crestal bone loss at implants has not yet been investigated. However, periodontal disease is thought to be a risk factor for implant loss [12]. The present cohort of patients with moderate periodontitis received systematic periodontal pre-treatment for 4 to 6 years on a 3-month recall schedule. Whether these results also apply to periodontally healthy patients and patients with advanced periodontal disease remains questionable.

In summary, due to insufficient data, many questions regarding the correlation between gingival phenotype and long-term success of implants are still unanswered. It is not yet known which teeth are suitable for determining the gingival and periodontal phenotype. Maxillary central incisors, followed by lateral incisors and canines, had the greatest mean gingival thickness [28, 29]. The gingival phenotype does not seem to be influenced by age or gender, but studies have reported a higher prevalence of the thin gingival phenotype in females [18, 30]. It appears to be a population-level characteristic, as Asian individuals have a thin gingival phenotype compared to Caucasians [31]. In addition, whether tooth shape predicts gingival phenotype is not clear, and the role played by buccal bone thickness has yet to be determined [19, 32].

Because of these unanswered questions, well-designed controlled clinical trials with low risk of bias will be necessary to determine the effects of gingival phenotype on the long-term success of implants. In particular, a more reliable, objective, and reproducible method for measuring soft tissue thickness and crestal bone level needs to be established. In future clinical studies, an intraoral ultrasound device with high-resolution probe could be a promising method for visualizing crestal bone level and soft tissue dimensions at implants [33, 34]. The sonographic examination can provide a better differentiation of the thickness and width of the soft tissue and clarify its individual influence on soft tissue inflammation and crestal bone loss.

## Conclusion

The results reported here for this long-term cohort study of patients with moderate periodontal disease treated on a recall schedule should be interpreted cautiously due to the small patient sample. However, crestal bone loss at implants during the first 12 months of functional loading was significantly higher in patients with gingival phenotype 1. After 1 year, crestal bone loss did not correlate with the gingival phenotype. The implants of patients with gingival phenotype 2 had significantly greater PD and higher risk of peri-implantitis. Further prospective long-term studies will be required to investigate the determination of gingival phenotype as a prognostic tool for predicting crestal bone loss at implants.

## Data Availability

The datasets used and/or analysed during the current study are available from the corresponding author on reasonable request.
